# Hybrid Multimodal Feature Fusion with Multi-Sensor for Bearing Fault Diagnosis

**DOI:** 10.3390/s24061792

**Published:** 2024-03-11

**Authors:** Zhenzhong Xu, Xu Chen, Yilin Li, Jiangtao Xu

**Affiliations:** 1 College of Aerospace and Civil Engineering, Harbin Engineering University, Harbin 150001, China; 2 School of Reliability and Systems Engineering, Beihang University, Beijing 100191, China; 3 School of Foreign Languages, Beijing Institute of Technology, Beijing 102488, China

**Keywords:** multimodal feature fusion, multi-sensor, PCA, ResNet, SVM, ensemble learning

## Abstract

Aiming at the traditional single sensor vibration signal cannot fully express the bearing running state, and in the high noise background, the traditional algorithm is insufficient for fault feature extraction. This paper proposes a fault diagnosis algorithm based on multi-sensor and hybrid multimodal feature fusion to achieve high-precision fault diagnosis by leveraging the operating state information of bearings in a high-noise environment to the fullest extent possible. First, the horizontal and vertical vibration signals from two sensors are fused using principal component analysis, aiming to provide a more comprehensive description of the bearing’s operating condition, followed by data set segmentation. Following fusion, time-frequency feature maps are generated using a continuous wavelet transform for global time-frequency feature extraction. A first diagnostic model is then developed utilizing a residual neural network. Meanwhile, the feature data is normalized, and 28 time-frequency feature indexes are extracted. Subsequently, a second diagnostic model is constructed using a support vector machine. Lastly, the two diagnosis models are integrated to derive the final model through an ensemble learning algorithm fused at the decision level and complemented by a genetic algorithm solution to improve the diagnosis accuracy. Experimental results demonstrate the effectiveness of the proposed algorithm in achieving superior diagnostic performance with a 97.54% accuracy rate.

## 1. Introduction

In contemporary industrial production environments, rotating machinery stands as one of the most prevalent pieces of mechanical equipment [1], with bearings serving as crucial components within such machinery. Faults in bearings can result in a decrease in technical indicators, substantial economic losses, and even significant safety incidents [2,3,4,5]. The majority of faults in rotating machinery stem from bearing issues [6]. Hence, timely and accurate identification of bearing faults holds immense importance in mitigating the occurrence of accidents [7]. The operational status of a bearing cannot be adequately captured by a single sensor due to variations in sensor monitoring locations and environmental interferences. Moreover, the operational environment of bearings frequently experiences disruptions from shock, wear, and corrosion, leading to changes in the bearing surface geometry and therefore to non-stationary and non-linear impulse responses on damaged contact surfaces [8]. Extracting and identifying fault characteristics pose significant challenges. Thus, it is imperative to address the non-stationary and non-linear attributes of vibration signals captured from multiple sensors to extract fault characteristics effectively and develop a high-precision fault diagnosis model based on these features.

In a practical working environment, it is difficult to diagnose bearing faults directly. Sensors can capture digital signals, such as vibration signals, reflecting the condition of the bearing. Signal processing and analysis enable the determination of the bearing’s condition [9,10]. During operation, the bearing generates vibration signals characterized by non-stationarity and non-linearity. Traditionally, signal features are extracted using methods such as empirical modal decomposition (EMD) [11] and wavelet transform (WT). EMD is primarily an empirical method. However, challenges such as modal aliasing arise during the signal decomposition process, limiting the applicability of EMD [12]. WT divides the signal’s frequency band into multiple scales, facilitating feature extraction from both the time and frequency domains. This approach has demonstrated effectiveness in practical applications [13]. However, the above methods inevitably lose fault information during the process; consequently, this paper proposes a multimodal feature fusion method based on multi-sensors in order to maximize the retention and extraction of fault information.

In real industrial environments, multiple sensors are commonly deployed. The fusion of signals or extracted features from these sensors constitutes a pivotal step preceding fault diagnosis. Lupea et al. [14] extracted signal features from both time and frequency domains and used them in the classification process to solve the proposed multi-fault detection problem. Ye et al. introduced a multi-level feature fusion network for integrating vibration signals, yielding promising outcomes [15]. Chen et al. employed sparse autoencoders to fuse signals from multiple sensors and utilized deep belief networks for fault diagnosis [16]. Zhu et al. employed wavelet packet transform and multi-weight singular value decomposition for extracting time-frequency features. They employed a support vector machine (SVM) classifier [17]. Shao et al. utilized denoising autoencoders and contractive autoencoders to learn features. Subsequently, they employed locality-preserving projection for feature fusion, enhanced feature quality, and finally applied softmax for fault diagnosis [18]. Wang et al. introduced a three-stage multimodal feature fusion approach for integrating vibration and torque signals. They coupled this with an attention-based multidimensional concatenated convolutional neural network for fault diagnosis [19]. Buchaiah et al. employed a random forest for feature selection. They utilized 14 feature extraction techniques to extract 2D features and employed a SVM classifier for fault diagnosis [20]. Wang et al. applied principal component analysis (PCA) to fuse multidimensional features, characterizing the operating condition of rolling bearings [21].

In summary, it is challenging to combine signals from multiple sensors and perform feature fusion to extract information that characterizes the operational status of the bearing. Furthermore, integrating the extracted features and leveraging diverse classifiers for a final diagnosis represents a crucial and challenging phase. In this paper, a fault diagnosis algorithm based on multi-sensor and hybrid multimodal feature fusion is proposed. Ultimately, the algorithm’s effectiveness is validated through comprehensive experimental results. The main contributions of this paper include the following:

(1) The fusion of horizontal vibration signals (HVS) and vertical vibration signals (VVS) from a multi-sensor in a feature layer yields dual-channel data. This approach maximizes the integration of feature information from both sensors, thereby enhancing the robustness and generalization capabilities of the algorithm.
(2) Initially, the dual-channel data is consolidated into a single-channel dataset. Continuous wavelet transform (CWT) is employed to extract global time-frequency feature information, generating time-frequency feature maps for training the residual neural network (ResNet). Simultaneously, time-frequency feature indexes are extracted post-normalization to obtain key indexes, facilitating training of the SVM. Utilizing global time-frequency features and key indexes for model training enhances the algorithm’s learning capability.
(3) Ensemble learning is employed to achieve decision-level fusion. The genetic algorithm (GA) is combined to address the multi-objective optimization model for obtaining weight parameters for the ResNet and SVM models. This integration harnesses the strengths of both base models, resulting in a diagnostic model with superior classification accuracy.

The remainder of the paper is organized as follows: The theoretical background, including multimodal feature fusion, time-frequency feature extraction, residual neural networks, support vector machines, and genetic algorithms, is briefly introduced in Section 2. Section 3 provides a description of the proposed diagnosis method in detail. In Section 4, the experimental results are analyzed and discussed. Finally, the conclusions are given in Section 5.

## 2. Theoretical Background

### 2.1. Multimodal Feature Fusion

PCA is a widely utilized algorithm for data dimensionality reduction. It recombines the original correlated variables into new variables that are uncorrelated with each other, aiming to retain as much information from the original variables as possible [22]. The information in the data is primarily captured by the variance, where a higher variance indicates more information content. The specific calculation steps are outlined below:

(1) Data preprocessing;
(2) Compute the matrix of correlation coefficients between variables, denoted as $R=\left(r_{st}\right),s,t=1,2,\ldots ,n$;
(3) Determine the eigenvalues $\lambda_{i}$ and corresponding eigenvectors $e_{i}=\left(e_{i1},e_{i2},\ldots ,e_{ip}\right),\left|\left|e_{i}\right|\right|=1$ for R;
(4) Compute the variance contribution Vcr and the cumulative variance contribution ratio Cvcr for the first l principal components as follows:
  (1) $$Vcr=\frac{\lambda_{i}}{\sum_{i=1}^{n}\lambda_{i}}$$
  (2) $$Cvcr=\frac{\sum_{i=1}^{l}\lambda_{i}}{\sum_{i=1}^{n}\lambda_{i}}$$
(5) Select the first l principal components $F_{1},F_{2},\ldots ,F_{l}$ based on the following cumulative variance contribution ratio:
  (3) $$F_{k}=e_{k1}X_{1}+e_{k2}X_{2}+\cdots +e_{kn}X_{n},\;k=1,2,\ldots ,l$$
  where $F_{i}$ is uncorrelated with $F_{j}\left(i\neq j;i,j=1,2,\ldots ,l\right)$ and $ar\left(F_{i}\right)>var\left(F_{j}\right),\;i<j$.

### 2.2. Time-Frequency Feature Extraction

#### 2.2.1. Continuous Wavelet Transform

WT, introduced by Gilles [23], is a signal processing technique designed for analyzing non-smooth, non-linear vibration signals. Similar to the Fourier transform, it employs a family of wavelet functions to represent the signal. Let us assume the function $\psi \in L^{2}\left(R\right)\cap L^{1}\left(R\right),\hat{\psi }\left(0\right)=0$. ψ is translated and scaled to generate the following set of functions:

(4) $$\psi_{a,b}\left(t\right)={\left|a\right|}^{-\frac{1}{2}}\psi \left(\frac{t-b}{a}\right),a,b\in R,a\neq 0$$

where $\psi_{a,b}$ represents an analytic or continuous wavelet; ψ is a fundamental wavelet; a is a scaling factor; and b is a translation factor.

CWT for an arbitrary function $f\left(t\right)\in L^{2}\left(R\right)$ is as follows:

(5) $$W_{f}\left(a,b\right)=<f,\psi_{a,b}>={\left|a\right|}^{-1/2}\int f\left(t\right)\bar{\psi \left(\frac{t-b}{a}\right)dt}$$

where $\bar{\psi \left(t\right)}$ represents the complex conjugate of $\psi \left(t\right)$; $<f,\psi_{a,b}>$ denotes the inner product; $W_{f}\left(a,b\right)$ denotes the coefficients of the wavelet function for a given scale factor a and translation factor b; a and b are continuous variables, and when the WT is performed in this manner, it is referred to as CWT.

In this study, CWT is employed to convert the vibration signals into time-frequency feature maps. The process is as follows:

(1) $f_{s}$ represents the sampling frequency, $F_{c}$ is the wavelet center frequency, and the actual center frequency corresponding to a is $F_{a}=F_{c}\times f_{s}/a$;
(2) Let totalsca*l* denote the length of the scale sequence during WT of the signal and c be a constant. The scale sequence takes the form $\frac{c}{totalscal},\ldots ,\frac{c}{totalscal-1},\frac{c}{4},\frac{c}{2},c$;
(3) According to step (1), c/toalscal corresponds to the actual frequency of $f_{s}/2$, thus $c=2\times F_{c}\times totalscal$. The scale sequence t can be calculated based on step (2);
(4) After determining the wavelet base and scale, the wavelet coefficient $W_{f}\left(a,b\right)$ is computed according to the principle. Following step (1), the scale sequence is converted into a frequency sequence f, which is then combined with the time sequence t to obtain time-frequency feature maps.

#### 2.2.2. Time-Frequency Indexes Extraction

When a fault occurs in the bearing, changes are observed in both time- and frequency-domain signals. Time-domain signals primarily undergo alterations in amplitude and distribution, while frequency-domain signals exhibit new frequency components and changes in spectral convergence. In the work of this paper, a total of 28 time-frequency indexes are selected, comprising 15 time-domain indexes and 13 frequency-domain indexes. These time-frequency characteristic indexes can comprehensively reflect the statistical characteristics of the bearing at the time of fault, thus helping to establish a fault diagnosis model.

As shown in Table 1 and Table 2, parameters $p_{1}$, $p_{12}–p_{15}$ primarily reflect changes in signal amplitude in the time domain, while parameters $p_{2}$, $p_{6}–p_{11}$ mainly describe the distribution of the signal in the time domain. Parameters $p_{3}–p_{5}$ mainly indicate the distribution of the energy in the time domain, while parameter $p_{16}$ primarily represents vibration energy in the frequency domain. Parameters $p_{17}–p_{19},\;p_{21}$, $p_{25}–p_{28}$ primarily describe changes in spectral power convergence, while parameters $p_{20}$, $p_{22}–p_{24}$ indicate positional changes in frequency [24].

The formulas for calculating the time domain characteristic indexes are shown in Table 1:

**Table 1:** Parameters of time domain characteristic indexes.

$p_{1}=\frac{\sum_{n=1}^{N}x\left(n\right)}{N}$	$p_{2}=\sqrt{\frac{\sum_{n=1}^{N}{\left(x\left(n\right)-p_{1}\right)}^{2}}{\left(N-1\right)}}$
$p_{3}={\left(\frac{\sum_{n=1}^{N}\sqrt{\left|x\left(n\right)\right|}}{N}\right)}^{2}$	$p_{4}=\sqrt{\frac{\sum_{n=1}^{N}{\left(x\left(n\right)\right)}^{2}}{N}}$
$p_{5}=\max\left|x\left(n\right)\right|$	$p_{6}=\frac{\sum_{n=1}^{N}{\left(x\left(n\right)-p_{1}\right)}^{3}}{\left(N-1\right){p_{2}}^{3}}$
$p_{7}=\frac{\sum_{n=1}^{N}{\left(x\left(n\right)-p_{1}\right)}^{4}}{\left(N-1\right){p_{2}}^{2}}$	$p_{8}=\frac{p_{5}}{p_{3}}$
$p_{9}=\frac{p_{5}}{p_{3}}$	$p_{10}=\frac{p_{4}}{\frac{1}{N}\sum_{n=1}^{N}\left|x\left(n\right)\right|}$
$p_{11}=\frac{p_{5}}{\frac{1}{N}\sum_{n=1}^{N}\left|x\left(n\right)\right|}$	$p_{12}=max\;x\left(n\right)$
$p_{13}=min\;x\left(n\right)$	$p_{14}=\left|p_{12}\right|+\left|p_{13}\right|$
$p_{15}=\left|p_{1}\right|$	

$x\left(n\right)$ is a time series, n=1,2,…,N. N is the number of data points.

The formulas for calculating the frequency domain characteristic indexes are shown in Table 2:

**Table 2:** Parameters of frequency domain characteristic indexes.

$p_{16}=\frac{\sum_{k=1}^{K}s\left(k\right)}{K}$	$p_{17}=\frac{\sum_{k=1}^{K}{\left(s\left(k\right)-p_{16}\right)}^{2}}{K-1}$
$p_{18}=\frac{\sum_{k=1}^{K}{\left(s\left(k\right)-p_{16}\right)}^{3}}{K{\left(\sqrt{p_{17}}\right)}^{3}}$	$p_{19}=\frac{\sum_{k=1}^{K}{\left(s\left(k\right)-p_{16}\right)}^{4}}{K{p_{17}}^{2}}$
$p_{20}=\frac{\sum_{k=1}^{K}f_{k}s\left(k\right)}{\sum_{k=1}^{K}s\left(k\right)}$	$p_{21}=\sqrt{\frac{\sum_{k=1}^{K}{\left(f_{k}-p_{20}\right)}^{2}s\left(k\right)}{K}}$
$p_{22}=\sqrt{\frac{\sum_{k=1}^{K}{f_{k}}^{2}s\left(k\right)}{\sum_{k=1}^{K}s\left(k\right)}}$	$p_{23}=\sqrt{\frac{\sum_{k=1}^{K}{f_{k}}^{4}s\left(k\right)}{\sum_{k=1}^{K}{f_{k}}^{2}s\left(k\right)}}$
$p_{24}=\sqrt{\frac{\sum_{k=1}^{K}{f_{k}}^{2}s\left(k\right)}{\sum_{k=1}^{K}s\left(k\right)\sum_{k=1}^{K}{f_{k}}^{4}s\left(k\right)}}$	$p_{25}=\frac{p_{21}}{p_{20}}$
$p_{26}=\frac{\sum_{k=1}^{K}{\left(f_{k}-p_{20}\right)}^{3}s\left(k\right)}{K{p_{21}}^{3}}$	$p_{27}=\frac{\sum_{k=1}^{K}{\left(f_{k}-p_{20}\right)}^{4}s\left(k\right)}{K{p_{21}}^{4}}$
$p_{28}=\frac{\sum_{k=1}^{K}{\left(s\left(k\right)-p_{20}\right)}^{\frac{1}{2}}s\left(k\right)}{K\sqrt{p_{17}}}$	

$s\left(k\right)$ is the spectrum, k=1,2,…,K. K is the number of spectral lines, and $f_{k}$ is the frequency value of the Kth spectral line.

### 2.3. Residual Neural Network

**Figure 1:**
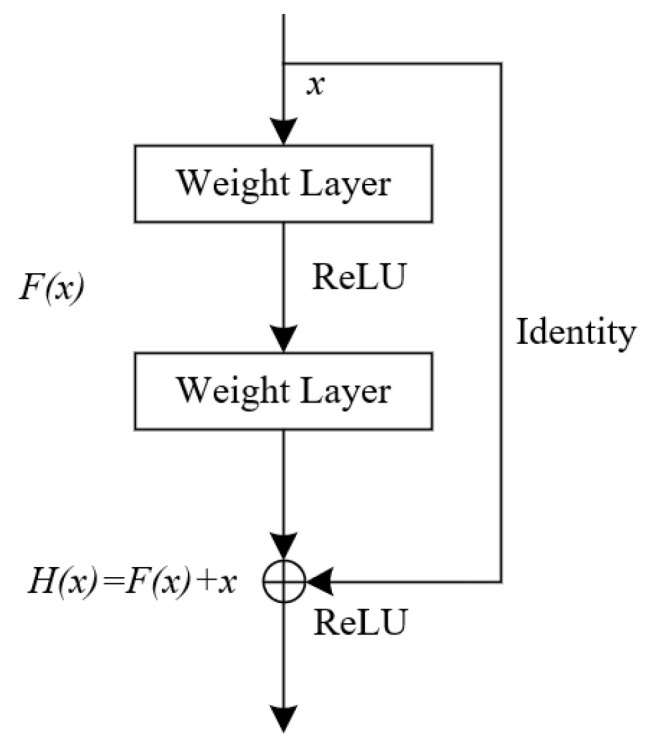
Residual block.

To address the issue of gradient disappearance encountered in traditional convolutional neural networks (CNN) as depth increases, He [25] introduced the ResNet. The key innovation of this network lies in the incorporation of residual blocks. The structure of the residual block is shown in Figure 1, which directly connects the residual block to the forward neural network. The forward neural network contains a convolutional layer, a batch normalization layer, and a Relu activation function layer. The input of the residual block is x, the output is $H_{x}$, and the forward neural network layer is $F_{x}$, $F_{x}=H_{x}-x$. For instance, in the case of a forward neural network layer with two weight layers, $F_{x}$ and x are element-wise summed up during direct concatenation, ensuring equal dimension mapping.

(6) $$\left\{\begin{array}{c} y=F\left(x,\left\{W_{i}\right\}\right)+x\\ F=W_{2}\sigma \left(W_{1}x\right) \end{array}\right.$$

where $F\left(x,\left\{W_{i}\right\}\right)$ is the residual mapping; F is the residual function; W is the weight parameter; and σ is the activation function.

When the dimensions of the mappings within the residual block are dissimilar, a linear mapping is performed on x to ensure dimensional alignment. Subsequently, the outputs of the linear mapping and the neural network layers are added together while preserving the dimensions.

(7) $$\left\{\begin{array}{c} y=F\left(x,\left\{W_{i}\right\}\right)+W_{s}x\\ F=W_{2}\sigma \left(W_{1}x\right) \end{array}\right.$$

where $W_{s}$ is the mapping weight of the input x.

ResNet offers enhanced optimization and improved accuracy compared to traditional convolutional neural networks, particularly as network depth increases. Different types and sizes of data can benefit from various configurations of ResNet, such as ResNet-18, ResNet-34, ResNet-50, ResNet-101, and ResNet-152.

### 2.4. Support Vector Machine

**Figure 2:**
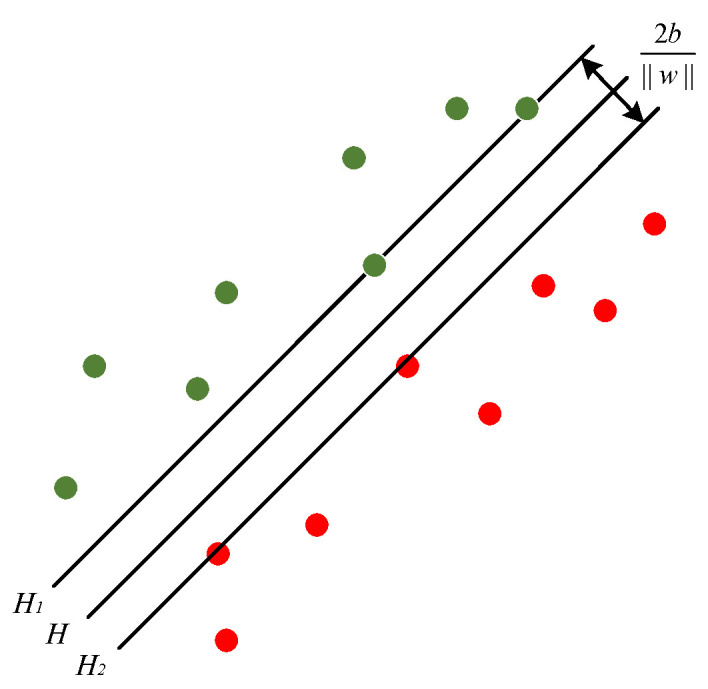
Optimal classification hyperplane.

SVM is a machine learning algorithm grounded in statistical theory, primarily employed for classification tasks [26]. Fault diagnosis based on time-frequency feature indexes inherently presents a challenge as a small-sample and nonlinear classification problem. SVM typically exhibits superior generalization to small-sample data compared to Back Propagation (BP), Extreme Learning Machines (ELM), Long Short-Term Memory (LSTM), and Deep Belief Network (DBN), which may suffer from overfitting. While SVM leverages kernel methods to address nonlinear classification issues, BP, LSTM, and DBN necessitate intricate architectures and parameter tuning to handle nonlinear relationships. SVM offers enhanced interpretability relative to BP and ELM, with the added benefit of avoiding local minima due to their convex optimization nature. LSTM excels at processing temporal information, yet in this paper, time-frequency feature indexes are extracted from temporal signals, which diverges from temporal information. In summary, for the specific problem addressed in this paper, SVM demonstrates a pronounced advantage. Its fundamental concept revolves around identifying the optimal classification hyperplane by maximizing the margin between the hyperplane and the classification data, as depicted in Figure 2:

Let the input data be $x_{i}=\left(i=1,2,\ldots ,M\right)$, with M representing the number of samples. For the binary classification problem, the classification label $y\epsilon \left\{-1,\;+1\right\}$. For linearly separable data, the corresponding hyperplane is defined as follows:

(8) $$f_{x}=w^{T}x+b=0$$

where w represents the M-dimensional weight vector and b is the bias scalar. These two parameters determine the distance between the hyperplane and the origin. Maximization of the margin between different classes is achieved by solving the following optimization problem:

(9) $$max\frac{2b}{{\left|\left|w\right|\right|}^{2}}$$

For cases where linear separability is not possible, a soft-spaced support vector machine is implemented by introducing the slack variable $\varepsilon_{i}\geq 0$ and the penalty factor C as follows:

(10) $$min\frac{1}{2}{\left|\left|w\right|\right|}^{2}+C\sum_{i=1}^{n}\varepsilon_{i}$$

For multi-classification problems involving N classes of samples, $N\left(N-1\right)/2$ standard SVM classifiers are trained, and the final result is based on the predictions of each classifier.

In SVM, the kernel function is employed to compute the similarity or inner product between input vectors, expressing the relationship between data in the feature space. The Radial Basis Function (RBF) is chosen as the kernel function due to its suitability for high-dimensional data and nonlinear mapping. The expression of the RBF kernel function is given by the following equation:

(11) $$k\left(x_{i},x_{j}\right)=exp\left(-\gamma \left|\left|x_{i}-x_{j}\right|\right|\right)$$

where $x_{i},x_{j}$ represent the input vector and center vector, respectively; $\left|\left|x_{i}-x_{j}\right|\right|$ denotes the Euclidean distance; and γ is a monotonic function that takes a small value when $x_{i}$ is far from $x_{j}$.

### 2.5. Genetic Algorithm

GA is an adaptive global optimization search technique inspired by nature’s genetic mechanisms and biological evolution, employing binary genetic coding [27]. Assuming that allele $\Gamma =\left\{0,1\right\}$ and individual space $H_{L}={\left\{0,1\right\}}^{L}$. Reproduction comprises crossover and mutation. The optimization steps are shown below:

(1) Determine the population size N, crossover probability $P_{c}$, mutation probability $P_{m}$, and termination criterion. Randomly generate N individuals as the initial population $X\left(0\right)$ and set an algebraic counter t←0.
(2) Calculate the fitness of individuals in $X\left(t\right)$.
(3) Select M/2 pairs of matrices from $X\left(t\right)$ using a selection operator, where M≥N.
(4) Perform crossover among the selected pairs to create M intermediate individuals according to $P_{c}$.
(5) Apply mutations to the M intermediate individuals according to $P_{m}$ to obtain M candidate individuals.
(6) Select N individuals from the M candidates based on fitness to form the new generation population $X\left(t+1\right)$.
(7) If the termination criterion has been satisfied, output the individual with maximum fitness in $X\left(t+1\right)$ as the optimal solution; otherwise, t←t+1 and return to (5).

## 3. Proposed Method

**Figure 3:**
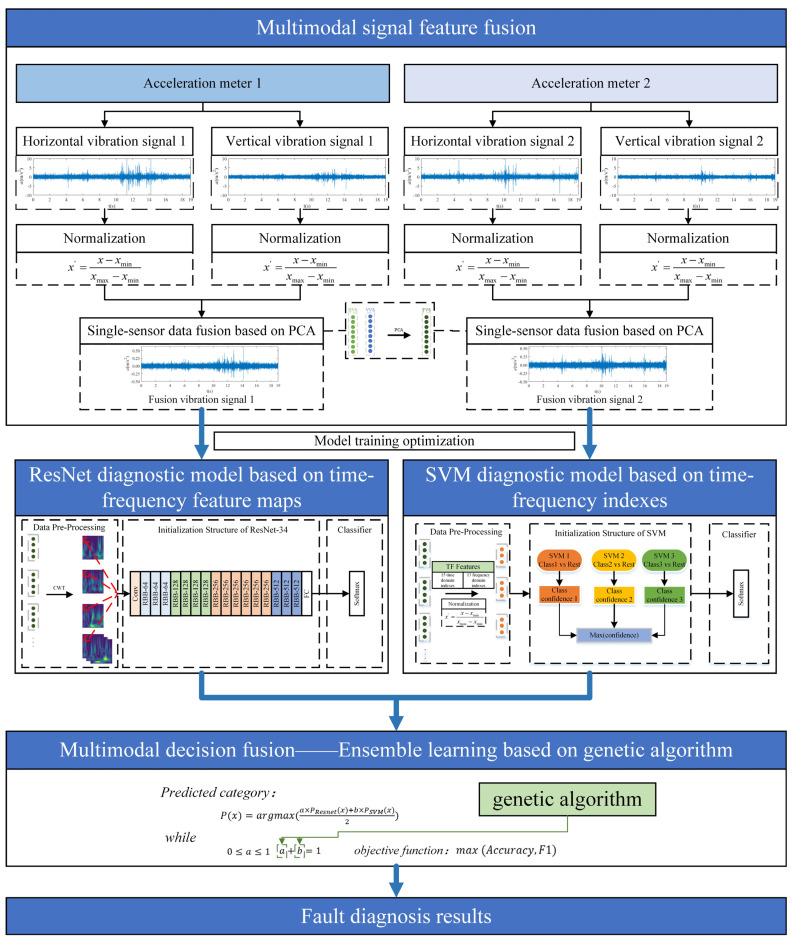
Fault diagnosis algorithm based on multi-sensor and hybrid multimodal feature fusion.

The proposed fault diagnosis algorithm leverages multi-sensor and hybrid multimodal feature fusion, as depicted in Figure 3. The model framework comprises the following three primary components: multimodal signal feature fusion, model training optimization, and multimodal decision fusion.

Initially, HVS and VVS from the bearing are captured by two sensors and normalized via min-max scaling. Subsequently, the vibration signals in both directions undergo fusion using min-max scaling and PCA. In the next stage, the fused feature signals (FVS) from the two sensors are partitioned into datasets. Time-frequency maps are generated using CWT, while a ResNet model is trained to establish a diagnostic model. Simultaneously, 28 time-frequency feature index parameters are extracted, normalized using min-max scaling, and used to train SVM for another diagnostic model. Finally, leveraging the base models obtained, GA optimizes the weight hyperparameters. The final diagnostic model is constructed using ensemble learning techniques, thereby enhancing the overall accuracy of the diagnostic system.

### 3.1. Multimodal Signal Feature Fusion

**Figure 4:**
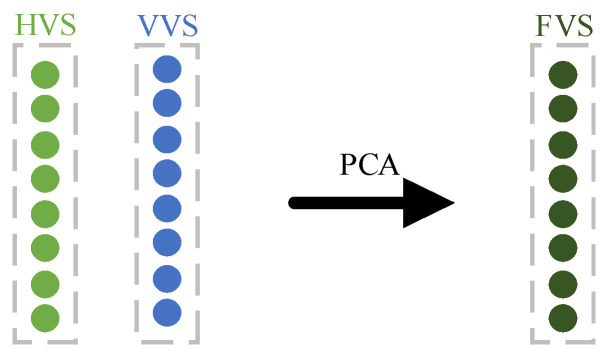
Single-sensor data fusion.

Two sensors, such as acceleration meters, are deployed at distinct locations on the test rig. Each sensor captures vibration signals across various modes in two directions: HVS and VVS. These signals, influenced by the sensor deployment positions and vibration directions, exhibit distinct fault characteristics. Initially, these signals are normalized using min-max scaling. Employing PCA facilitates data dimensionality reduction and enables the fusion of diverse modal signals from each sensor to yield FVS, as depicted in Figure 4.

### 3.2. Model Training Optimization

#### 3.2.1. ResNet Diagnostic Model Based on Time-Frequency Feature Maps

**Figure 5:**
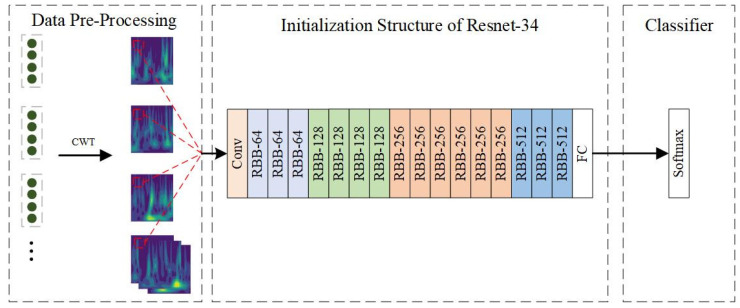
ResNet-34 diagnostic model flowchart.

After acquiring the FVS through PCA, the signal undergoes segmentation to generate a dataset based on the sampling frequency and interval. Subsequently, CWT is applied to analyze and process feature information across time and frequency domains, resulting in the generation of time-frequency maps from three channels. Finally, the ResNet-34 model is trained to establish the initial base classification model. The model architecture and steps are as depicted in Figure 5:

The loss function adopts the cross-entropy loss function, which is defined as shown in the following equation:

(12) $$L=\frac{1}{N}\underset{i}{\sum }L_{i}=-\frac{1}{N}\underset{i}{\sum }\sum_{c=1}^{M}y_{ic}log\left(p_{ic}\right)$$

where M is the number of categories; $y_{ic}$ denotes the sign function (0 or 1), taking 1 if the true category of sample i is equal to c and 0 otherwise; and $p_{ic}$ is the predicted probability of observing that sample i belongs to category c. The optimization algorithm used in this paper is stochastic gradient descent.

#### 3.2.2. SVM Diagnostic Model Based on Time-Frequency Indexes

**Figure 6:**
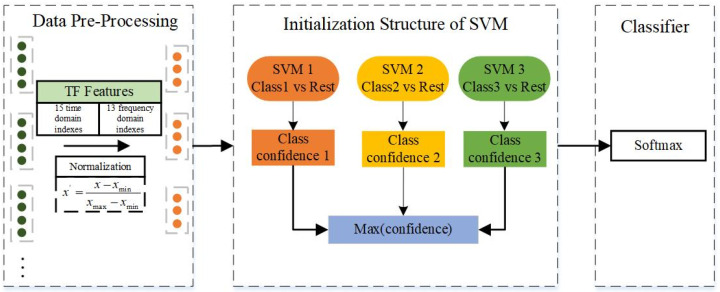
SVM diagnostic model flowchart.

On the alternate pathway, employing the time-frequency index formulas, 28 key indexes are computed for each data segment. Following normalization via min-max scaling, SVM is trained. The One-vs.-Rest strategy is adopted to enable multi-class classification, thereby yielding an additional base model. The model architecture and steps are outlined as depicted in Figure 6:

The dataset utilized in this study comprises high-dimensional, linearly indivisible data, prompting the adoption of the RBF as the kernel function due to its ability to handle such data structures effectively. The penalty factor C and the RBF parameter γ play pivotal roles in shaping the accuracy of the classification model. 

To optimize the SVM classification model, this paper employed the grid search method, a technique widely recognized for its effectiveness in determining optimal parameter values. Through systematic exploration within predefined ranges, the grid search method facilitates the identification of the most suitable values for C and γ, ensuring the creation of an accurate classification model.

### 3.3. Multimodal Decision Fusion

After training to obtain two base models, ResNet and SVM, set a weight parameter for each base model to combine their diagnostic results for the purpose of decision fusion. Define two weight coefficients a,b as shown in the following equation:

(13) 0≤a≤1

(14) a+b=1

(15) $$P\left(x\right)=argmax(\frac{a\times P_{Resnet}\left(x\right)+b\times P_{SVM}\left(x\right)}{2})$$

where $P\left(x\right)$ is the predicted category; $P_{Resnet}\left(x\right)$ is the probability of the category predicted by the ResNet model; $P_{SVM}\left(x\right)$ is the probability of the category predicted by the SVM model; a is the weight coefficient assigned to the ResNet model; and b is the weight coefficient assigned to the SVM model.

Table 3 shows the confusion matrix for the binary classification, which presents the complete evaluation of the classification results.

(16) $$Accuracy=\frac{TP+TN}{TP+FP+FN+TN}$$

(17) $$Precision=\frac{TP}{TP+FP}$$

(18) $$Recall=\frac{TP}{TP+FN}$$

(19) $$F1=\frac{2\times Precision\times Recall}{Precision+Recall}$$

GA is employed to optimize the weight hyperparameters a,b in the context of maximizing both accuracy and *F*1 score. This optimization task presents a multi-objective problem, where the objective function aims to balance the trade-off between accuracy and *F*1 score as follows:

(20) $$max\left(Accuracy,F1\right)$$

## 4. Case Study

**Figure 7:**
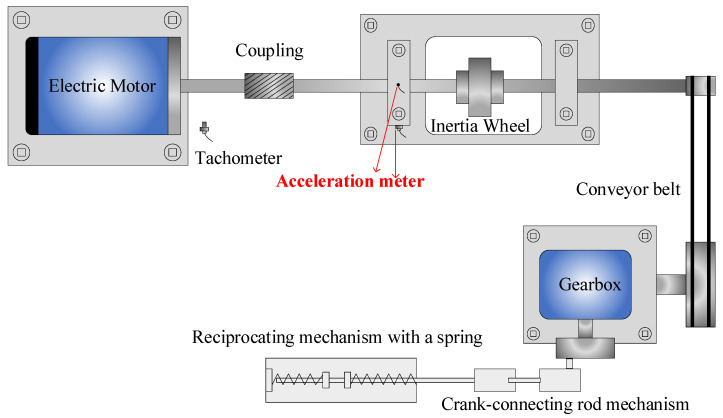
A schematic of the bearing test rig.

In order to evaluate the effectiveness of the proposed algorithm, experimental tests were conducted using the test rig depicted in Figure 7. The components of the test rig include an electric motor, an inertia wheel for applying radial load, a coupling, a conveyor belt drive mechanism, a conveyor belt, a crank-connecting rod mechanism, a gearbox, and a reciprocating mechanism with a spring. The bearing under investigation is mounted close to the motor side and is identified as a contact deep groove ball bearing (MB ER-10K). Three distinct types of faults, namely inner race fault (IF), outer race fault (OF), and rolling element fault (RF), were intentionally implanted in the bearing.

For fault implantation, localized cracks with a width and depth of 0.2 mm were introduced in the outer race groove, inner race groove, and ball surface of the bearing. Two acceleration meters (AM), specifically PCB Model 608A11, were strategically mounted at locations indicated in Figure 7. These meters, with a sensitivity of 10.2 (mV/ms^−2^)/100 (mv/g), played a crucial role in capturing vibration data essential for fault diagnosis. The rotational speed is 900 revolutions per minute. The acquisition card used in the experiment is the NI 9234, operating at a sampling frequency of 25.6 kHz. 

The raw signals of the three types of faults measured by the multi-sensor are shown in Table 4:

From Table 4, it is evident that the amplitude of time-domain signals varies significantly across different fault types. Typically, the signals exhibit distinct amplitudes: the RF signal displays the highest amplitude, followed by the IF signal, with the OF signal registering the smallest amplitude. Additionally, it is observed that the amplitude difference among measured signals in various directions for the same fault remains relatively minor.

### 4.1. Feature Layer Fusion

HVS and VVS from each of the multi-sensors are fused using PCA. The contribution of the two principal components after fusion is shown in Table 5:

From Table 5, it can be seen that for the three faults, the principal component 1 of fused vibration signals from the two sensors can cover 63–84% of the original information. This table can prove that the signals after PCA fusion well extract the fault feature information.

**Figure 8:**
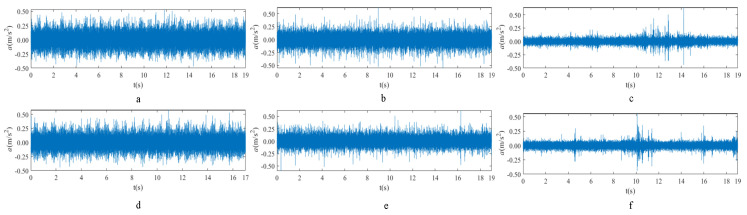
FVS. (**a**) AM 1 IF; (**b**) AM 1 OF; (**c**) AM 1 RF; (**d**) AM 2 IF; (**e**) AM 2 OF; (**f**) AM 2 RF.

In this paper, principal component 1 is chosen as FVS. The fused feature signal is shown in Figure 8:

In Figure 8, it is evident that following data normalization, the amplitudes predominantly fall within the range of −0.5 to 0.5. Notably, the amplitude ranges vary noticeably across the three different faults examined. Specifically, the amplitude of the IF signal exhibits a broader distribution, while that of the RF signal appears more concentrated. This distinction suggests varying fault characteristics within the analyzed signals. Furthermore, despite normalization, the overall waveform remains largely consistent with the original vibration signal, indicating the preservation of fundamental signal characteristics post-normalization.

### 4.2. Base Model Training Optimization

The dataset was obtained with a sampling frequency of 25.6 kHz. Each segment of FVS is sliced into intervals of 0.05 s, containing 1280 sample points. In total, 2240 segments are generated from the dataset. The dataset is divided into training and test sets according to an 8:2 ratio, resulting in 1792 segments allocated for training and 448 segments for testing.

#### 4.2.1. ResNet-34 Diagnostic Model

CWT is employed to process the datasets and generate time-frequency feature maps. Given that the fault frequencies predominantly lie within the low-frequency band, the vertical coordinate display range is constrained to 0–1000 Hz to capture the primary global feature information. This study utilizes the Gaussian wavelet as the wavelet function for the CWT, with a scale factor set to 256 to ensure comprehensive feature extraction across the signal spectrum.

**Figure 9:**
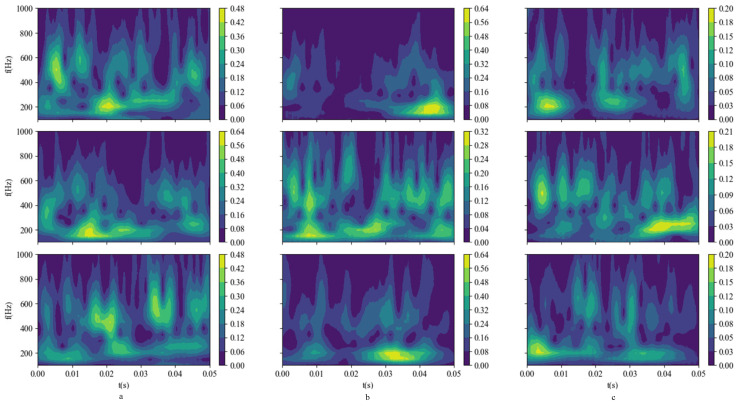
CWT time-frequency feature maps. (**a**) IF; (**b**) OF; (**c**) RF.

For illustration, consider a subset of three signals from each fault type. Their respective time-frequency feature maps are presented as depicted in Figure 9:

The ResNet model, comprising ResNet-18, ResNet-34, ResNet-50, ResNet-101, and ResNet-152 architectures, is employed for training on the extracted time-frequency feature maps. These models are evaluated to select the most effective diagnostic model for the task at hand. Table 6 provides a summary of the model parameters for each ResNet architecture.

The input image size is set to 200 × 200 pixels with three channels (RGB). Conv1 serves as a preprocessing convolutional layer, featuring a 7 × 7 convolutional layer with a stride of 2, followed by a batch normalization layer, and a 3 × 3 maximum pooling layer with a stride of 2. Subsequently, Conv2_x, Conv3_x, Conv4_x, and Conv5_x represent the residual structures, each comprising a variable number of residual blocks. The output of the final residual layer undergoes average pooling before entering the fully connected layer (FC) for classification. Finally, the softmax classifier outputs the probability values corresponding to the categories, yielding the recognition results.

**Figure 10:**
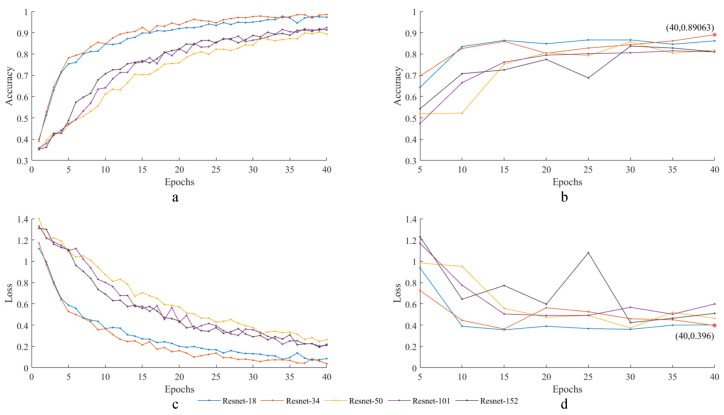
Train and test results (**a**) train accuracy; (**b**) test accuracy; (**c**) train loss; (**d**) test loss.

In this paper, the training iteration is 40 epochs, and the model effect is tested at five epoch intervals. The training and testing results are shown in Figure 10.

From Figure 10a,c, it is observed that the ResNet-34 model demonstrates faster convergence in terms of accuracy and loss on the training set, outperforming other models. From Figure 10b,d, the ResNet-34 model achieves an accuracy of 89.06% with a corresponding loss of 0.396 after 40 epochs of training, indicating superior performance compared to alternative models.

**Figure 11:**
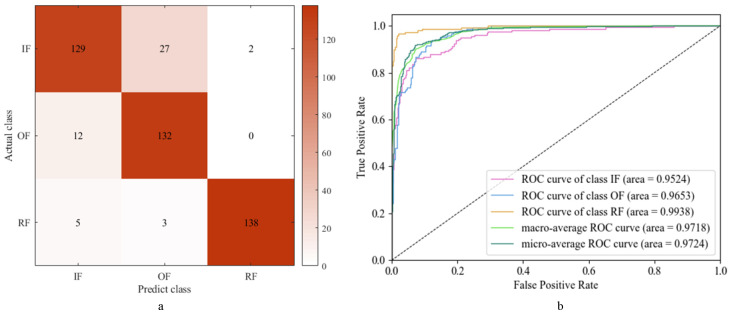
ResNet-34 diagnosis results: (**a**) confusion matrix; (**b**) ROC and AUC.

The classification results of the faults are shown in Figure 11, which reveal that the ResNet-34 model achieves a precision of 89.22%, a recall of 89.47%, and an *F*1 score of 0.8928. These metrics collectively demonstrate the model’s effectiveness in fault classification, with ResNet-34 showcasing superior performance in comparison to other models. Furthermore, the AUC values for all three fault types are observed to be close to one, indicating excellent classification performance across the board. Consequently, the ResNet-34 model emerges as a robust base model for subsequent ensemble learning diagnostic models.

#### 4.2.2. SVM Diagnostic Model

Utilizing the 28 time-frequency index calculation formulas, 28 time-frequency feature indexes are obtained for each piece of data. In order to visualize the expression of these time-frequency feature indicators for the features, the t-SNE method is used to visualize the high-dimensional data.

**Figure 12:**
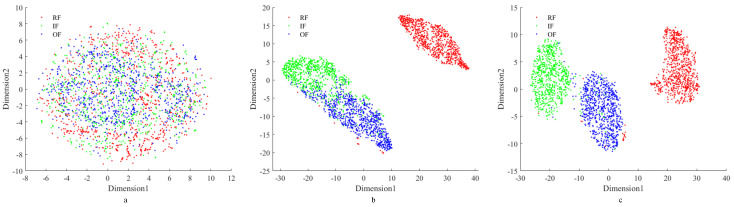
Two-dimensional visualization by t-SNE. (**a**) FVS; (**b**) time-frequency indexes; (**c**) time-frequency indexes after normalization.

Figure 12a demonstrates that FVS on a two-dimensional plane makes it challenging to distinguish between various types of data points effectively. However, Figure 12b reveals that after extracting 28 time-frequency indexes, clear differentiation among data points is observed, particularly with RF being notably distinct from IF and OF, while differentiation between IF and OF is less pronounced. In this study, min-max normalization is employed to normalize the time-frequency indexes. Figure 12c illustrates that after normalization, the three types of fault data points become highly distinguishable, effectively expressing the characteristic information of each fault type while minimizing the impact of noise. The enhanced distinguishability among fault types is crucial for accurate fault diagnosis and analysis.

In this study, RBF is employed as the kernel function for SVM to perform classification tasks. The classification effectiveness of the SVM model is primarily influenced by the following two key parameters: the penalty factor C and the parameter γ of the RBF kernel function. The values of the penalty factor C and the parameter γ of the RBF kernel function are shown in Table 7.

**Figure 13:**
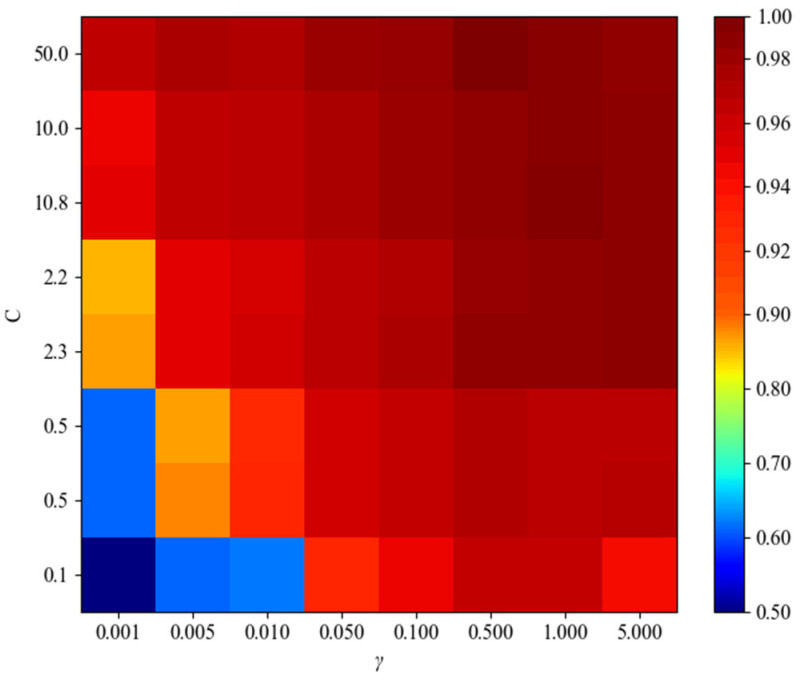
SVM classification accuracy.

In this paper, the grid search method is used to solve the penalty factor C and the parameter γ. The classification accuracy of the SVM model is shown in Figure 13.

The model classification accuracy is 95.76% when C is 50 and γ is 0.5, which is the best performance among the parameter combinations.

**Figure 14:**
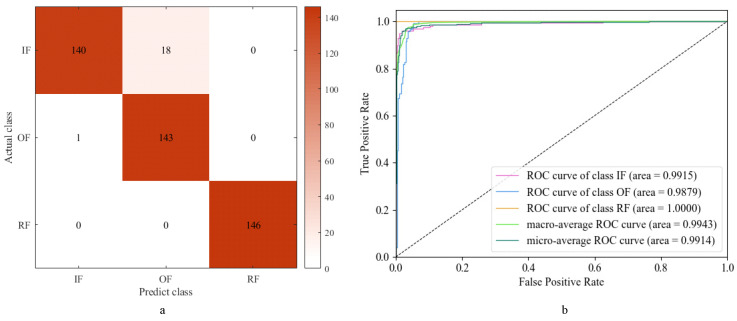
SVM diagnosis results: (**a**) confusion matrix; (**b**) ROC and AUC.

The results of fault diagnosis using the SVM model are presented in Figure 14a. The model demonstrates high precision (96.04%), recall (95.97%), and *F*1 score (0.9581), indicating its effectiveness in accurately classifying the three types of faults. Additionally, Figure 14b illustrates that the AUC values for the three fault types are close to one, underscoring the SVM’s robust classification performance across fault categories. These findings affirm the SVM’s superior classification efficacy in fault diagnosis, warranting its selection as another base model for subsequent ensemble learning diagnostic models.

### 4.3. Diagnostic Model Based on Ensemble Learning

In this study, ensemble learning techniques are employed to combine the diagnostic results of two base models through decision-level fusion, aiming to enhance the accuracy of fault diagnosis. The synthesis of diagnostic results is achieved using GA, which iteratively optimizes the weight coefficient values a,b assigned to the base models.

GA aims to maximize the accuracy and *F*1 score, treating them as fitness functions guiding the optimization process. A population size of 50 individuals is initialized, with a maximum population size capped at 100 to ensure computational efficiency. A mutation probability of 0.2 and a crossover probability of 0.7 are defined to introduce variability and exploration within the population.

The optimization process iterates over 1–15 generations, seeking to converge towards individuals with the highest fitness values, which represent the optimal weight coefficient values for combining the base models. By leveraging GA, the study seeks to identify the most effective combination of base models that maximizes diagnostic accuracy and *F*1 score, ultimately improving fault diagnosis performance.

**Figure 15:**
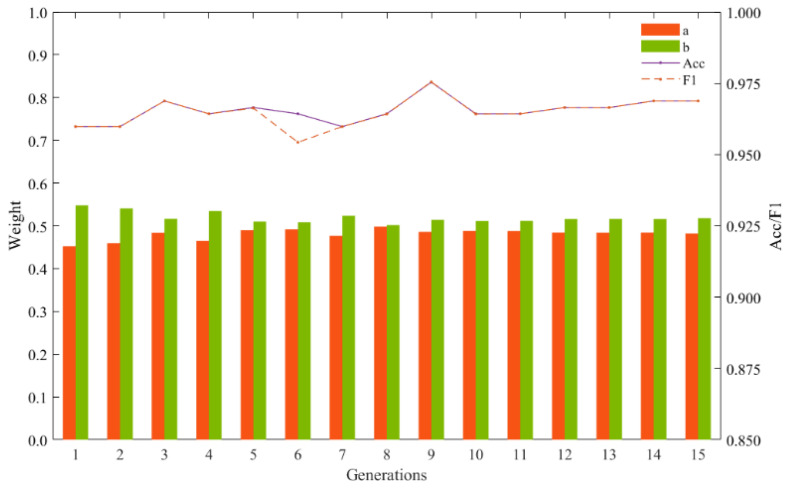
GA results.

According to the GA solution results in Figure 15, the accuracy and F1 of the model reached their maximum value when the population was iterated to the ninth generation and a was found to be 0.4859 and b was 0.5141. Therefore, these two values were used as weights for the base model ResNet-34 and the SVM diagnostic model.

**Figure 16:**
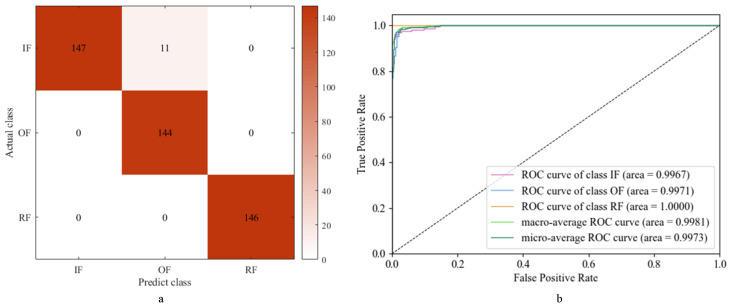
Ensemble model diagnosis results: (**a**) confusion matrix; (**b**) ROC and AUC.

Using this integrated learning model for the test set, the diagnostic results are obtained as shown in Figure 16.

The diagnostic results of the ensemble learning model for three types of faults are presented in Figure 16a. The model achieves an impressive accuracy rate of 97.54%, a precision rate of 97.63%, a recall rate of 97.68%, and an *F*1 score of 0.9757. Additionally, Figure 16b illustrates that the AUC values for the three fault types are very close to one, indicating excellent classification performance across fault categories.

As depicted in Figure 11, Figure 14, and Figure 16, distinguishing between IF and OF poses a challenge primarily due to the mechanical similarity between the inner and outer races. The operational faults occurring in the inner and outer races of the bearing result in a partial overlap of their spectral components. Additionally, factors such as sampling frequency, sensor placement, and noise interference further complicate the distinction between IF and OF.

As shown in Table 8, these evaluation metrics collectively demonstrate the superior performance of the ensemble learning model compared to the individual base models. The ultimate diagnostic model demonstrated an 8.48% and 1.78% enhancement in accuracy compared to the two foundational models, accompanied by improvements in the other three metrics to varying degrees. By combining the strengths of the ResNet-34 and SVM diagnostic models through ensemble learning, the model achieves a better diagnostic effect, effectively enhancing fault diagnosis accuracy and reliability.

## 5. Conclusions

The proposed fault diagnosis algorithm based on multi-sensor and hybrid multimodal feature fusion shows promise in improving diagnostic accuracy and credibility. The fusion of hybrid multimodal features is delineated into two stages. In the initial phase, vibration signals from distinct sensors in varying directions are amalgamated at the feature layer. Subsequently, in the second stage, diagnostic outcomes from diverse modal data sources are integrated at the decision layer employing ensemble learning techniques. By fusing features from the HVS and VVS using PCA, the algorithm extracts global time-frequency information through CWT and key index information using 28 time-frequency indexes. This approach addresses the non-stationary and non-linear characteristics of vibration signals and enhances the accuracy and credibility of diagnostic results.

The utilization of ResNet-34 and SVM models for fault diagnosis, followed by ensemble learning for decision layer fusion, further improves diagnostic performance. Despite the overall effectiveness of the proposed algorithm, misclassifications between IF and OF remain a challenge. Subsequent research should optimize the feature extraction technique, enhance the classifier structure, and enrich the model training with additional data. These steps are essential for effectively discerning between bearing IF and OF.

In conclusion, this paper presents an effective solution for leveraging multi-sensor vibration signals of different modes for fault diagnosis in high-noise environments. The proposed approach employs a two-stage fusion process involving the feature layer and the decision layer, resulting in an efficient fault diagnosis algorithm. The algorithm shows promise as a robust classifier, opening avenues for further refinement and optimization in future research endeavors.

## Figures and Tables

**Table 3:** Confusion matrix for the binary classification.

Total Population	Condition Positive	Condition Negative
Predicted condition positive	True positive (*TP*)	False positive (*FP*)
Predicted condition negative	False negative (*FN*)	True negative (*TN*)

**Table 4:** Vibration signal time domain.

AM	Fault Type	HVS	VVS
1	IF	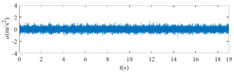	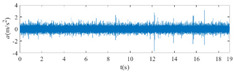
	OF	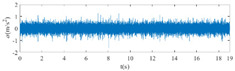	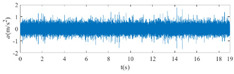
	RF	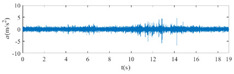	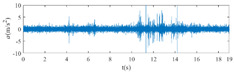
2	IF	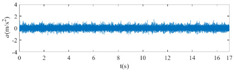	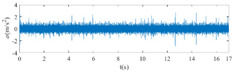
	OF	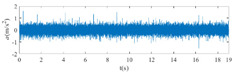	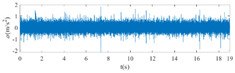
	RF	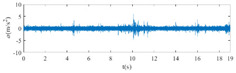	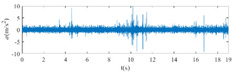

IF refers to inner race fault, OF to outer race fault, and RF to rolling element fault; AM refers to acceleration meter, HVS to horizontal vibration signals, and VVS to vertical vibration signals.

**Table 5:** PCA results.

AM	Fault Type	Principal Component 1	Principal Component 2
1	IF	0.83593733	0.16406267
	OF	0.63439911	0.36560089
	RF	0.81361577	0.18638423
2	IF	0.76050419	0.23949581
	OF	0.64756928	0.35243072
	RF	0.71948348	0.28051652

IF refers to inner race fault, OF to outer race fault, and RF to rolling element fault; AM refers to acceleration meter, HVS to horizontal vibration signals, and VVS to vertical vibration signals.

**Table 6:** ResNet model parameters.

Layer Name	Output Size	18-Layer	34-Layer	50-Layer	101-Layer	152-Layer
Input layer	--	(200,200,3)				
Conv1	112 × 112	7 × 7, 64, stride 2				
	56 × 56	bn, 3×3 max pool, stride 2				
Conv2_x	56 × 56	$\left[\begin{array}{c} 3\times 3,64\\ 3\times 3,64 \end{array}\right]\times 2$	$\left[\begin{array}{c} 3\times 3,64\\ 3\times 3,64 \end{array}\right]\times 2$	$\left[\begin{array}{c} 1\times 1,64\\ 3\times 3,64\\ 1\times 1,256 \end{array}\right]\times 3$	$\left[\begin{array}{c} 1\times 1,64\\ 3\times 3,64\\ 1\times 1,256 \end{array}\right]\times 3$	$\left[\begin{array}{c} 1\times 1,64\\ 3\times 3,64\\ 1\times 1,256 \end{array}\right]\times 3$
Conv3_x	28 ×28	$\left[\begin{array}{c} 3\times 3,128\\ 3\times 3,128 \end{array}\right]\times 2$	$\left[\begin{array}{c} 3\times 3,128\\ 3\times 3,128 \end{array}\right]\times 4$	$\left[\begin{array}{c} 1\times 1,64\\ 3\times 3,64\\ 1\times 1,256 \end{array}\right]\times 4$	$\left[\begin{array}{c} 1\times 1,64\\ 3\times 3,64\\ 1\times 1,256 \end{array}\right]\times 3$	$\left[\begin{array}{c} 1\times 1,64\\ 3\times 3,64\\ 1\times 1,256 \end{array}\right]\times 8$
Conv4_x	14×14	$\left[\begin{array}{c} 3\times 3,256\\ 3\times 3,256 \end{array}\right]\times 2$	$\left[\begin{array}{c} 3\times 3,256\\ 3\times 3,256 \end{array}\right]\times 6$	$\left[\begin{array}{c} 1\times 1,64\\ 3\times 3,64\\ 1\times 1,256 \end{array}\right]\times 6$	$\left[\begin{array}{c} 1\times 1,64\\ 3\times 3,64\\ 1\times 1,256 \end{array}\right]\times 23$	$\left[\begin{array}{c} 1\times 1,64\\ 3\times 3,64\\ 1\times 1,256 \end{array}\right]\times 36$
Conv5_x	7 × 7	$\left[\begin{array}{c} 3\times 3,512\\ 3\times 3,512 \end{array}\right]\times 2$	$\left[\begin{array}{c} 3\times 3,512\\ 3\times 3,512 \end{array}\right]\times 3$	$\left[\begin{array}{c} 1\times 1,64\\ 3\times 3,64\\ 1\times 1,256 \end{array}\right]\times 3$	$\left[\begin{array}{c} 1\times 1,64\\ 3\times 3,64\\ 1\times 1,256 \end{array}\right]\times 3$	$\left[\begin{array}{c} 1\times 1,64\\ 3\times 3,64\\ 1\times 1,256 \end{array}\right]\times 3$
Output layer	1 × 1	Average pool, 1000-d fc, softmax				

**Table 7:** Parameter values.

C		γ	
0.10	0.50	0.001	0.005
0.46	2.32	0.010	0.050
2.15	10.77	0.100	0.500
10.00	50.00	10.000	50.000

**Table 8:** Classification evaluation metrics.

Algorithm	Accuracy	Precision	Recall	*F*1
ResNet-34	0.8906	0.8922	0.8947	0.8928
SVM	0.9576	0.9604	0.9597	0.9581
The proposed ensemble model	0.9754	0.9763	0.9768	0.9757

## Data Availability

Data contained within the article.
